# An in vitro study evaluating the efficacy of a novel mount with torque control designed to tighten Osstell® transducers

**DOI:** 10.1002/cre2.734

**Published:** 2023-04-07

**Authors:** David Naughton, Erica Donnelly‐Swift, Ioannis Polyzois

**Affiliations:** ^1^ Department of Restorative Dentistry and Periodontology Dublin Dental University Hospital, Trinity College Dublin Ireland; ^2^ Department of Public Health and Primary Care Institute of Population Health, Trinity College Dublin Dublin Ireland; ^3^ Dublin Dental University Hospital, Trinity College Dublin Dublin Ireland

**Keywords:** dental implants, resonance frequency analysis

## Abstract

**Objectives:**

To evaluate the efficacy of a novel mount with torque control for tightening of Osstell® transducers and to determine the reliability of recorded ISQ measurements from implants placed in various bone densities.

**Material and Methods:**

Fifty‐six implants, comprising seven different implant types, were placed in eight polyurethane blocks representing D1, D2, D3, and D4 bone densities. Resonance frequency analysis (RFA) transducers were attached to each implant in four different ways: (a) hand tightening, (b) hand tightening with a SmartPeg Mount™, (c) hand tightening using the novel mount with torque control (SafeMount) and (d) tightening to 6 Ncm with a calibrated torque device. ISQ measurements were taken and a second operator repeated the measurements. Intraclass correlation coefficient (ICC) was calculated to assess the reliability of the measurements and linear mixed effects regression was employed to determine the effect explanatory variables had on ISQ values.

**Results:**

There was a statistically significant difference in ISQ values obtained by hand tightening transducers compared to the calibrated torque device *p* < .001, 95%(−2.89, −1.21) but not between any other tightening methods. There was excellent agreement between the two RFA devices (ICC 0.986) and between buccal and mesial measurements (ICC 0.977). For all transducer tightening methods there was excellent inter‐operator agreement in D1 and D2 (ICC > 0.8) but very poor agreement in D4 (ICC < 0.24). Bone density accounted for 36% of the variation in ISQ values, the implant for 11% and the operator for 6%.

**Conclusions:**

SafeMount, did not significantly improve the reliability of the RFA measurements when compared to the standard mount, but calibrated torque devices seem to have benefits when compared to tightening the transducers by hand. Results also indicate that the ISQ values should be interpreted with caution when measuring implant stability in poor quality bone regardless of the implant geometry.

## 1 INTRODUCTION

Assessment of implant stability in an accurate and noninvasive manner helps guide the loading protocol and provides information regarding the likely prognosis of a dental implant. Implants with poor primary stability, have higher failure rates as there is a significant association between primary and secondary stability (Meredith, 1998; Monje et al., 2019). Additionally, while implants with high stability can be provisionalised immediately, those with poorer stability may require a more conventional two‐stage approach resulting in longer treatment times for patients.

Measuring stability at various time points can be done using resonance frequency analysis (RFA). First described in 1996, Osstell® have manufactured several generations of devices to assess implant stability by RFA. A transducer, termed a SmartPeg, is attached to an implant and a specialized probe is placed near the SmartPeg, emitting a sinusoidal wave to gently bring the SmartPeg and implant to vibration. The measurement of this vibration is then expressed as an implant stability quotient (ISQ), a nonlinear scale of 1–100. Higher implant stability corresponds to a higher ISQ value and several factors can affect these readings. Bone density and the macro‐ and micro‐geometries of the implant have all been shown to have an effect (Comuzzi et al., 2021; Gehrke et al., 2015; Marquezan et al., 2012; Merheb et al., 2018; Novellino et al., 2017). The influence of SmartPeg tightening has only been scantly assessed to date.

An intimate fit of a SmartPeg would theoretically give the most accurate ISQ value of an implant by fully allowing for measurement of the vibration of the implant itself. A SmartPeg not sufficiently tightened would move somewhat independent of the implant and an overtightened SmartPeg may damage the connection with the implant, also resulting in inaccurate readings. Osstell® recommends a SmartPeg to be “finger tight.”

A study by Geckili et al. recommended SmartPegs to be tightened to 5–8 Ncm (Geckili et al., 2015) but a similar study by Kästel et al. reported that there is no need for standardization of tightening forces as all their investigated values, (2–11 Ncm) resulted in accurate ISQ readings (Kästel et al., 2019). Salatti et al. in agreement with Geckili et al. reported that there was a need for standardization of tightening forces, but recommended tightening the Smart Pegs™ to 10–17 Ncm of torque (Salatti et al., 2019).

At present, there is disagreement as to the optimal torque value that should be applied to SmartPegs and they are either tightened by hand or by using a plastic mount supplied by Osstell®. In a bid to introduce a degree of torque control, Osstell® developed a novel plastic mount termed “SafeMount.” Once a certain amount of torque is applied with this mount, a tactile and audible click is emitted. Osstell® reports this to be happening at 4–4.5 Ncm.

The aim of the current study was to evaluate the effect that different transducer tightening protocols might have on the accuracy of ISQ values of implants placed in artificial bone blocks. An effort was also made to determine the reliability of recorded ISQ measurements from implants with varying macro and microgeometries placed in various bone densities.

## 2 MATERIALS AND METHODS

### 2.1 Implant insertion

**Figure 1:**
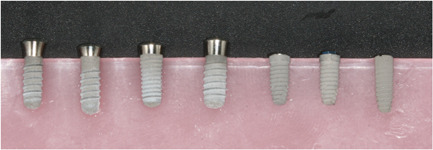
The seven types of implant used.

Seven types of implants were used in this study (Figure 1):

1. Standard 4.1 × 10 mm SLA (Institute Straumann AG®)
2. Standard Plus 4.1 × 10 mm SLA (Institute Straumann AG®)
3. Tapered Effect 4.1 × 10 mm SLA implant (Institute Straumann AG®)
4. Standard 4.8 × 10 mm SLA (Institute Straumann AG®)
5. BNST 4.0 × 10 mm internal hexagonal connection implant (Zimmer Biomet®)
6. BOET 4.0 × 10 mm external hexagonal connection (Zimmer Biomet)
7. Ankylos™ C/X 3.5 × 11 mm (Dentsply® Sirona)

**Figure 2:**
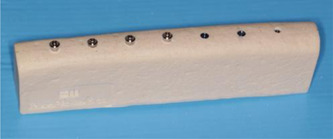
Bone block with all seven types of implant inserted.

Eight resin polyurethane bone blocks (BoneModels™) were used in this study. Each block was of uniform density and represented either D1, D2, D3, or D4 bone densities. Two blocks of each density were used. One of each implant type was inserted into each block with uniform spacing. In total, 7 implants were placed per block resulting in 56 implants (Figure 2). All implants were placed according to the implant manufactures' protocols. Implant osteotomies in D1 bone blocks were completed with a bone tap. Implant osteotomies in D4 bone blocks were undersized to achieve stability.

### 2.2 Bench top set up and measurements

Osstell® SmartPeg transducers were paired to implants as per Osstell's recommendation for each implant and all measurements were taken in a buccal and mesial direction.

**Figure 3:**
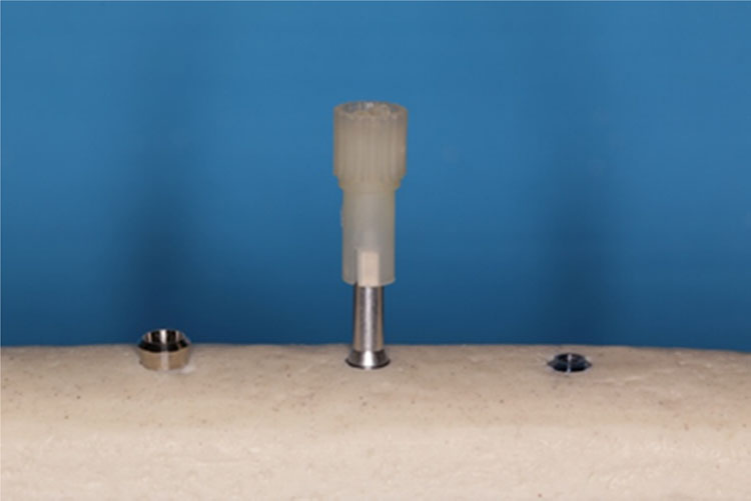
Safemount on a SmartPeg™ inserted into a Biomet 3i® internal connection implant.

Transducers were attached to each implant in four different ways:

- Group 1: Hand tightened by using gauze (handtighten).
- Group 2: Hand tightened with the standard plastic mount supplied with SmartPegs (plastic mount).
- Group 3: Hand tightened with the novel (SafeMount) (Figure 3).
- Group 4 (control): 6 Ncm of standardized force with a calibrated torque wrench (Tohnichi®). The National Metrology Laboratory calibrated the device (6 Ncm).

Bone blocks were secured to a bench top with a vice. A SmartPeg™ was attached to each implant in a randomized order by means of an online randomizer in each of the four ways. Two Osstell® devices were used throughout to record ISQ values (Osstell IDx and Osstell ISQ). All initial measurements were repeated by the second Osstell® device. All measurements were taken with the probe tip approximately 2 mm and 90° to the SmartPeg in a buccal and mesial direction. ISQ values were recorded onto an Excel (Microsoft Excel v16.55) spreadsheet. A second operator then repeated the above described bench top set up and measurements.

### 2.3 Statistical analysis

Descriptive statistics were used to express mean ISQ values by torquing method. Medians of ISQ values by implant type were compared using a Kruskal–Wallis test as values were not normally distributed (Kolmogorov–Smirnov test) when all bone densities were included and also when D4 was omitted. Dunn's multiple comparisons post hoc test was used when *p* < .05. Different implant systems were only compared for one of the four mounting groups (group 4) as this is generally considered the optimal tightening force. An average measures intraclass correlation coefficient (ICC) was also calculated to determine how well the measurements from the two Osstell devices, the two investigators and the two directions, resembled each other. ICC results were classified according to Koo and Li guidelines (Koo & Li, 2016). These statistical analyses were performed with IBM SPSS software v27.

**Figure 4:**
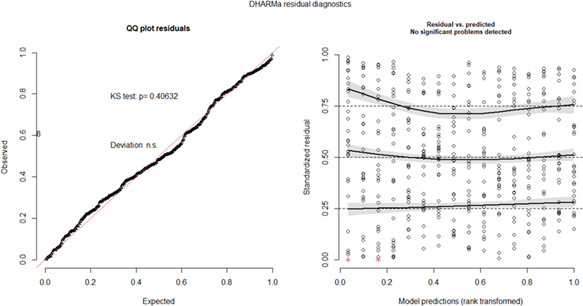
QQ plot and residual diagnostics for the hierarchical (multilevel/mixed) regression model.

Linear mixed effects regression was employed to determine the effect that explanatory variables had on ISQ readings. Model complexity was determined by examining change in deviance and information criterion—Akaike's Information Criterion and Bayesian information criterion. The final model selected followed the principle of parsimony. Models were developed using Statistical Software: R (version 4.1.2) (Team RC, 2021) and R Studio (Team R, 2021). Models were further developed using packages lme4 (Bates et al., 2015) and lmerTest (Kuznetsova et al., 2017). *p* values displayed for the model are based Satterthwaite's method of approximation. Model diagnostics were checked using a simulation‐based approach on scaled residuals from the R package DHARMa (Hartig, 2021). Package emmeans was used for post hoc comparisons (Lent et al., 2021). Model diagnostics were performed on simulated residuals. Results from the outlier test and the Kolmogorov–Smirnov test was nonsignificant (*p* > .5). A QQ‐plot of the standardized residuals illustrated a linear pattern thus indicating that residuals satisfied assumptions are satisfied (Figure 4).

## 3 RESULTS

Overall, 1792 ISQ measurements were performed. The mean ISQ values and the spread of ISQ values were similar for each of the four tightening groups when all bone densities were included (Table 1).

**Table 1:** Table of mean ISQ values by SmartPeg tightening group.

SmartPeg tightening method	Mean ISQ, SD
Hand tightening with gauze	64.17 ± 12.99
Plastic SmartPeg mount	65.89 ± 13.57
SafeMount	66.04 ± 14.33
Calibrated torque wrench (6 Ncm)	66.22 ± 14.12

Abbreviation: ISQ, implant stability quotient.

**Figure 5:**
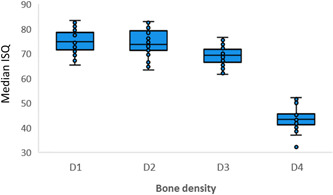
Boxplot of implant stability quotient values by bone density.

As expected, a large difference in ISQ values could be seen based on bone density when all four tightening groups were included (Figure 5).

**Figure 6:**
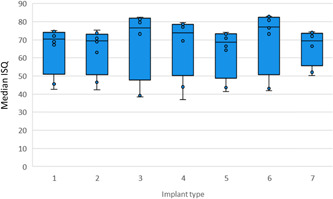
Boxplot of implant stability quotient values by implant type in all bone densities with SmartPegs torqued to 6 Ncm. Type 1: Straumann Standard, 2: Straumann Aesthetic Plus, 3: Straumann Tapered Effect, 4: Straumann wide body, 5: BNST 4.0 × 10 mm internal hexagonal connection implant (Zimmer Biomet®), 6: BOET 4.0 × 10 mm external hexagonal connection (Zimmer Biomet®), 7: Ankylos™ C/X 3.5 × 11 mm (Dentsply®).

**Figure 7:**
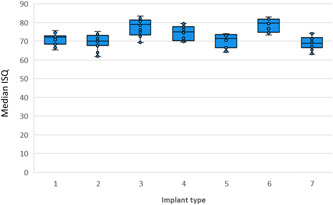
Boxplot of implant stability quotient values by implant type in D1, D2, and D3 with SmartPegs torqued to 6 Ncm. Type 1: Straumann Standard, 2: Straumann Aesthetic Plus, 3: Straumann Tapered Effect, 4: Straumann wide body, 5: BNST 4.0 × 10 mm internal hexagonal connection implant (Zimmer Biomet®), 6: BOET 4.0 × 10 mm external hexagonal connection (Zimmer Biomet®), 7: Ankylos™ C/X 3.5 × 11 mm (Dentsply®).

To assess the specific influence of the implant on ISQ values and spread, ISQ values were arranged to compare the seven implant types when SmartPegs were tightened only with the calibrated torque device (6 Ncm of torque). The difference in ISQ values obtained from the various implants was not statistically significant when all bone densities were included in the analysis (*p* = .361) (Figure 6) but did reach significance when measurements from D4 bone were omitted (*p* < .001) (Figure 7). The most significant differences were observed between implants 2 versus 3, 2 versus 6 (*p* < .001) and 5 versus 6 (*p* < .001).

ICC was excellent between operators (0.995), devices (0,986) and direction of Osstell® probe (buccal and mesial) (0.977). Furthermore, ICC values were calculated between operators for each SmartPeg tightening method in each type of bone density. There was excellent agreement when measuring in D1 and D2 bone between examiners. In D3, agreement was good but it became very poor in D4 bone (Table 2).

**Table 2:** Table of ICC values between operator 1 and 2 for each type of bone density by SmartPeg torquing method.

Bone density	Hand torque with gauze	Plastic torque wrench	SafeMount	6 Ncm of torque
D1	0.830	0.811	0.977	0.944
D2	0.934	0.972	0.972	0.983
D3	0.791	0.767	0.762	0.803
D4	0.112	0.192	0.238	0.039

Abbreviation: ICC, intraclass correlation coefficient

Mixed linear effects regression analysis showed a statistically significant difference between ISQ values in the hand tightening group (*p* < .001) and the values in the control group. No statistically significant differences were detected between the ISQ values from the other two groups and the control. Additionally, the differences between the ISQ values measured in D3 (*p* = .006) and D4 (*p* < .001) bone were statistically significant to the ones measured in D1 (Table 3).

**Table 3:** Table demonstrating the influence of SmartPeg tightening method and bone density on mean ISQ values.

Characteristics	Coef	95% CI	*p* Value
Torqueing method			
6 Ncm torque	Ref		
Hand tightening with gauze	–2.05	(–2.89, –1.21)	<.001
Plastic torque wrench	–0.33	(–1.17, 0.51)	.444
SmartMount	–0.19	(–1.03, 0.66)	.668
Bone density			
1	Ref		
2	–0.27	(–3.88, 3.35)	.889
3	–5.90	(–9.52, –2.28)	.006
4	–31.50	(–35.11, –27.89)	<.001

Abbreviation: ISQ, implant stability quotient.

Examination of random effects indicate that approximately 10.36% of the total variation of random effects is due to the nested effect of the implant within each bone density per bone block. Approximately 36.34% of the total random variation was due to the implant within each bone density. The effect for Implant alone accounts for approximately 11.23% of the total variation of random effects and the effect of operator alone accounts for 6.06% (Table 4).

**Table 4:** Table demonstrating the percentage variance due to random effects.

Random effects	Variance	% of total
Bone block:density:implant	3.00	10.36
Density:implant	10.52	36.34
Implant	3.25	11.23
Operator	1.76	6.08
Residual variance	10.42	35.99

## 4 DISCUSSION

Both operators involved in the study showed excellent agreement between their measurements, while using two different Osstell® devices. This finding is in agreement with previous evidence. Norton, recently compared the same two Osstell® devices used in the study herein, (Norton, 2019). He evaluated ISQ values in 210 implants at placement and 3 months after insertion. In the mesiodistal direction, he reported an ICC value of 0.97 between ISQ values from each device. Of note, there was a lower agreement for bucco‐palatal measurements, with an ICC value of 0.71. Norton noted that the Osstell® IDx appeared to give several “false” readings of 35 ISQ units on several occasions. The same trend was not observed in the study herein.

Measurements were similar in D1 and D2 bone, displayed a slight drop in D3 bone and had a significant fall in D4 bone. This is in agreement with the current literature where the influence of bone density on ISQ values has been demonstrated. In a systematic review, Marquezan et al. reported moderate to strong correlations between bone density, measured by Hounsfield units, and ISQ values (Marquezan et al., 2012). Similarly, Ivanova et al. recently showed a significant positive linear correlation between ISQ values and bone density as measured by bone biopsies from a trephine bur before the implant osteotomy (Ivanova et al., 2021).

When testing for reliability of measurements depending on SmartPeg tightening method, the highest agreement with the control group was obtained using the SafeMount (ICC 0.994) and the lowest, was obtained using the hand torquing method (ICC 0.978). Linear mixed effects regression analysis demonstrated a statistically significant difference between ISQ values obtained from hand tightening SmartPegs compared to the control. No statistically significant difference was seen between the standard SmartPeg mount or the novel SafeMount and the control. These findings agree with the findings of earlier studies where hand torqueing, resulted in lower ISQ values (Geckili et al., 2015; Salatti et al., 2019).

There are only a handful of studies assessing the influence of the tightening torque of SmartPegs on ISQ. One study concluded that all their investigated torque values of 2–11 Ncm were appropriate (Kästel et al., 2019). Whereas, the other two study groups recommended specific values of 5–8 Ncm (Geckili et al., 2015) and 10–17 Ncm (Salatti et al., 2019). They reported that too low a torque value reduced the ISQ value. Osstell® recommends SmartPegs to be tightened to 4–5 Ncm which they report to be “finger tight.” This present study used 6 Ncm as the control value. This value allowed for agreement with both the Kästel et al. and Geckili et al. studies.

The effect of the implant design on ISQ is well studied and can affect ISQ values. All past studies assessing SmartPeg torque values used only one implant system each and little information was provided on the specific implants. This current study aimed to address this by using a variety of different implants replicating the clinical reality. This is of interest as the anatomy of the intaglio surface of each type of implant and its possible effect on the RFA measurements hasn't been previously studied. In theory, the intimacy of the fit of the connection of the SmartPeg would affect the ISQ value.

Given that it seems that bone density had an effect on the reliability of measurements a linear mixed effects model was performed to compare the effect of bone density to other factors such as the operator and the implant. The implant within each bone density accounted for the largest amount of variance in measurements at approximately 36%. The implant itself accounted for 11% and the operator for 6%. The nested effect of the implant within bone density per bone block accounted for 10% of the variation. This shows bone density to have the largest influence on the ISQ measurements.

Although the ICC was slightly higher for the SafeMount group compared to the standard SmartPeg mount group, this difference was not significant. The greatest advantage of the SafeMount is possibly for standardization of the use of Osstell® SmartPegs for research purposes and for long term monitoring of changes in ISQ. There may also be a role for their use with users who are inexperienced with Osstell® SmartPegs, to ensure they have applied a sufficient amount of force when tightening the SmartPeg. In clinical practice, there may also be a change in operators between the implant surgery and restoration and nonaccurate measurements might falsely alarm the operator regarding possible bone loss.

A limitation of this study is that the statistical power did not allow for a meaningful comparison between the different individual designs of implants. There were no similar previous studies to base a priori power calculation on and post‐hoc power calculations are often reported to be conceptually flawed with misleading results (Gelman, 2019; Senn, 2002). A further limitation is that only two operators were involved in hand tightening the SmartPegs. As demonstrated by Pelegrine et al. there can be significant differences between different operators however, both operators in this study, overall, had excellent agreement with each other (Pelegrine et al., 2020). Furthermore, the two operators were male. A broader range of operators, including female operators, would allow for a greater generalizability of these results.

In conclusion, the plastic SmartPeg mount currently supplied by Osstell® and the novel SafeMount, both performed similarly to the control. In general, transducer tightening methods seem to have an impact on the reliability of ISQ values. Results also indicate that the ISQ values should be interpreted with caution when measuring implant stability in poor quality bone regardless of the implant geometry.

## AUTHOR CONTRIBUTIONS

Ioannis Polyzois contributed to the conception and design of the study. David Naughton organized the database. Erica Donnelly‐Swift performed the statistical analysis. David Naughton wrote the first draft of the manuscript. Ioannis Polyzois and Erica Donnelly‐Swift wrote sections of the manuscripts. All authors contributed to the article and approved the submitted version.

## CONFLICT OF INTEREST STATEMENT

The authors declare no conflict of interest.

## Data Availability

The raw data supporting the conclusions of this article are available by the corresponding author upon reasonable request.
